# Comparing different stimulus configurations for population receptive field mapping in human fMRI

**DOI:** 10.3389/fnhum.2015.00096

**Published:** 2015-02-20

**Authors:** Ivan Alvarez, Benjamin de Haas, Chris A. Clark, Geraint Rees, D. Samuel Schwarzkopf

**Affiliations:** ^1^Institute of Child Health, University College LondonLondon, UK; ^2^Institute of Cognitive Neuroscience, University College LondonLondon, UK; ^3^Wellcome Trust Centre for Neuroimaging, University College LondonLondon, UK; ^4^Experimental Psychology, University College LondonLondon, UK

**Keywords:** population receptive field modeling, pRF, retinotopy, fMRI, visual cortex, primary visual cortex (V1), stimulus design

## Abstract

Population receptive field (pRF) mapping is a widely used approach to measuring aggregate human visual receptive field properties by recording non-invasive signals using functional MRI. Despite growing interest, no study to date has systematically investigated the effects of different stimulus configurations on pRF estimates from human visual cortex. Here we compared the effects of three different stimulus configurations on a model-based approach to pRF estimation: size-invariant bars and eccentricity-scaled bars defined in Cartesian coordinates and traveling along the cardinal axes, and a novel simultaneous “wedge and ring” stimulus defined in polar coordinates, systematically covering polar and eccentricity axes. We found that the presence or absence of eccentricity scaling had a significant effect on goodness of fit and pRF size estimates. Further, variability in pRF size estimates was directly influenced by stimulus configuration, particularly for higher visual areas including V5/MT+. Finally, we compared eccentricity estimation between phase-encoded and model-based pRF approaches. We observed a tendency for more peripheral eccentricity estimates using phase-encoded methods, independent of stimulus size. We conclude that both eccentricity scaling and polar rather than Cartesian stimulus configuration are important considerations for optimal experimental design in pRF mapping. While all stimulus configurations produce adequate estimates, simultaneous wedge and ring stimulation produced higher fit reliability, with a significant advantage in reduced acquisition time.

## Introduction

The visual receptive field (RF) of a neuron is the area of the visual field upon which stimulation causes a response. Due to the retinotopic organization of cortical visual field maps, selective responses to visual stimulation can be studied non-invasively in humans at a coarser resolution using functional magnetic resonance imaging (fMRI) (for a review, see Wandell and Winawer, 2011). In visual field mapping studies carried out in the 1990s, responses to systematic stimulation of the visual field were used to define the organization of retinotopic maps in human cerebral cortex (Engel et al., 1994, 1997; Sereno et al., 1995; DeYoe et al., 1996). By calculating the phase-difference between a periodic visual stimulus presentation and fMRI signals recorded from occipital cortex, it was possible to estimate the position in visual space eliciting maximal responses at each cortical location (voxel). This in turn allowed the localization and delineation of different retinotopic visual field maps according to their polar angle and eccentricity representations (Wandell et al., 2007; Bridge, 2011).

While these methods are effective in localizing and delineating different retinotopic areas, they do not allow us to probe the underlying characteristics of the RFs of individual neurons, such as the tuning curve, size, or shape. To date, such characteristics can only be directly measured by invasive electrophysiological recordings. However, the signal captured by fMRI methods pools together the hemodynamic responses associated with activation of hundreds of thousands of neurons in a single voxel, so in visually responsive cortex the signal from specific locations will reflect a complex aggregate of the properties of individual RFs. As visual cortex is organized retinotopically, at the spatial scale of fMRI methods these responses will reflect characteristics of many neurons encoding a common area of visual space, hence the concept has arisen of a population receptive field (pRF). Originally coined in electrophysiology (Victor et al., 1994), a pRF refers to the aggregate properties of a large number of neighboring neurons, that by their topographic organization share common features, such as responsiveness to stimulation to a given visual field location. Early work studied cortical pRFs indirectly; either by varying stimulus size to infer the cortical image point spread (Tootell et al., 1997), or by accounting for the responses of sampled voxels beyond the stimulus cycling frequency using a data-fitting approach to estimate the relative proportion of time a given voxel was active during stimulation, or duty cycle (Smith et al., 2001). Later work developed an explicit model-based approach, where the area of visual space that elicits responses in a single voxel is modeled as a Gaussian function (Larsson and Heeger, 2006). To do this, a simulated time series of the hypothetical fMRI signals given a certain receptive field profile is compared against the experimentally observed time series. By comparing multiple combinations of receptive field properties (e.g., location, spread) with the observed data, a best-fitting pRF model is obtained for each cortical location. Model frameworks for estimation of pRFs now integrate an array of visual stimulation scenarios and model components, allowing not only visual map localization but also the estimation of other parameters such as pRF size and cortical magnification factors (Dumoulin and Wandell, 2008; Harvey and Dumoulin, 2011).

Model-based approaches to estimating pRF characteristics allow the study of pRF dynamics (Haak et al., 2012; Zuiderbaan et al., 2012), the properties of striate (Kok and de Lange, 2014; Verghese et al., 2014), and extra-striate visual areas (Amano et al., 2009; Winawer et al., 2010; Harvey and Dumoulin, 2011; Dumoulin et al., 2014) as well as abnormal visual field representations in developmental disorders (Schwarzkopf et al., 2014) and disease (Baseler et al., 2011; Hoffmann et al., 2012; Brewer and Barton, 2014; Papanikolaou et al., 2014). Despite the growing popularity of this approach, it is currently unclear whether there is an optimal stimulus design for pRF estimation, and whether there are inherent biases in certain stimulus configurations.

In the seminal paper by Dumoulin and Wandell (2008) a combination of stimuli are used, including a polar angle wedge and eccentricity ring stimuli, as traditionally used in phase-encoded retinotopic mapping (Sereno et al., 1995; DeYoe et al., 1996; Engel et al., 1997), with the addition of a size-invariant bar traversing the visual field linearly along multiple orientations. The use of a moving bar aperture has been widely adopted, with many studies implementing it alone (Harvey and Dumoulin, 2011; Zuiderbaan et al., 2012; Brewer and Barton, 2014; de Haas et al., 2014; Dumoulin et al., 2014; Papanikolaou et al., 2014; Schwarzkopf et al., 2014; Verghese et al., 2014). Despite this ready adoption, it remains unclear whether the use of a size-invariant bar aperture is optimal for pRF mapping. Binda et al. (2013) examined whether stimulus design created inherent biases in receptive field estimation in the neighborhood of scotomas. When examining both bar and multifocal stimuli, that is, a stimulus where checkerboards segments are presented in pseudo-randomized groups in order to reduce the correlation between any given pair of segments, they concluded that both bar and multifocal designs biased pRF estimates when a virtual scotoma was introduced. Notably, these biases were reduced by actively modeling the scotoma in a multifocal design, but not in a bar stimulus design, pointing to biases in model estimation interacting with stimulus choice.

Stimulus optimization has previously been reported in phase-encoded retinotopic mapping methods, with various configurations proposed depending on the experimental question at hand (e.g., Tootell et al., 1997; Slotnick and Yantis, 2003). More recently, multifocal stimuli have been used as a way of boosting precision in retinotopic localization (Buracas and Boynton, 2002; Hansen et al., 2004; Vanni et al., 2005; Henriksson et al., 2012), but with significantly reduced explanatory power (Ma et al., 2013). In pRF modeling, Binda et al. (2013) report a similar reduction in power of a multifocal stimulus compared to sweeping bars, albeit with the additional finding that bars returned larger pRF sizes compared to the multifocal stimulus, highlighting a trade-off in pRF stimulus design that has yet to be studied systematically. In the present study, we investigated the effects of two design variables; eccentricity scaling and the use of stimuli defined in polar (simultaneous wedge and ring) vs. Cartesian (bars) coordinates on pRF model estimates. Multi-aperture stimulus designs, which include multifocal stimuli and the novel simultaneous wedge and ring stimulus used here, have not been previously explored in terms of pRF mapping efficiency, despite a theoretical advantage for them in terms of efficiency with which the pRF is measured. By definition, single aperture stimuli, such as sweeping bars, can only measure one stimulus dimension at a time. During the same time it takes the bar to sweep along a full set of directions, a traditional wedge or ring stimulus can collect several cycles thus potentially increasing the reliability with which that dimension is mapped. By further combining wedge and ring stimuli and presenting them simultaneously, both polar angle and eccentricity coordinates can theoretically be estimated multiple times. The added value of a multi-aperture display can therefore improve the sampling rate of polar coordinate-defined stimuli toward increased stimulus efficiency. Here we compared three stimulus configurations to assess their relative efficiency for pRF mapping; a size-invariant sweeping bar as implemented in Dumoulin and Wandell (2008), a similar version of the stimulus that scaled logarithmically with eccentricity and a new polar coordinate-based simultaneous wedge and ring stimulus.

## Materials and methods

### Participants

Three authors (IA, BdH, DSS) and five naive adults (6 males and 2 females, age range: 23–36) took part in the study. All participants were healthy, had normal or corrected-to-normal visual acuity, and provided written informed consent. The study was approved by the local ethics committee, in compliance with the Declaration of Helsinki.

### Visual stimulation

Stimuli were generated in MATLAB (v8.0, Mathworks Inc., Natick, MA, USA) using Psychtoolbox (v3.0, Brainard, 1997; Pelli, 1997) and displayed on a back-projection screen in the bore of the magnet via an LCD projector. Participants viewed the back-projection via a mirror mounted on the head coil. The visual display subtended a maximum visual angle of 16° eccentricity from fixation for two participants and 9° eccentricity for a further six participants, in order to test the effects of viewing distance on estimates of stimulus eccentricity. All further stimuli measures are given for the large display area; the measures for the smaller display area were simply scaled down.

#### General description of stimuli

**Figure 1:**
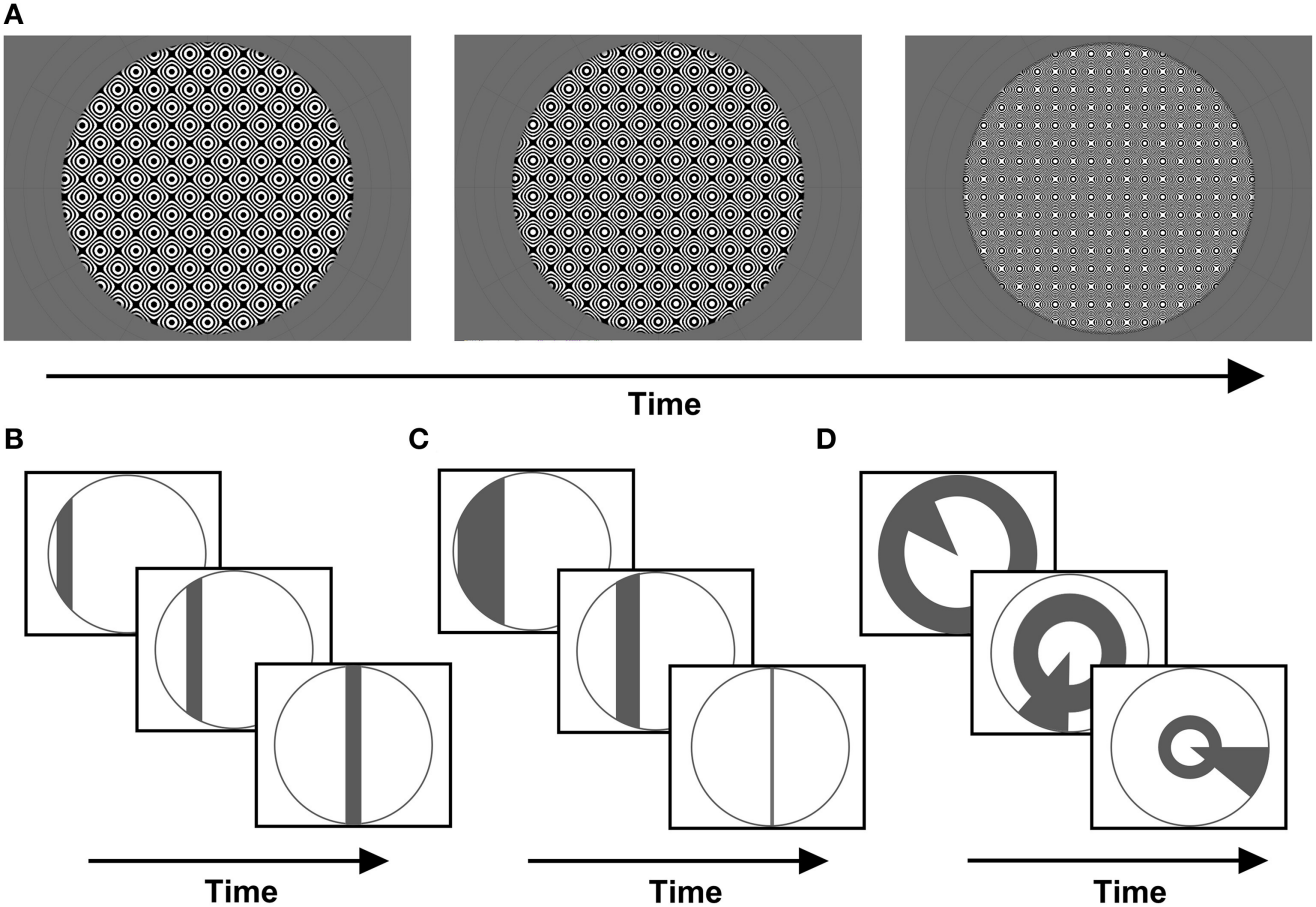
**(A)** Example frames from the stimulus carrier, a checkerboard-like, luminance-modulated pattern varying in spatial frequency, described in full in the methods section. Stimuli were presented with either **(B)** size-invariant bar apertures, **(C)** bars logarithmically-scaled with eccentricity, or **(D)** a simultaneous “wedge and ring” aperture, cycling at different frequencies and scaled logarithmically with eccentricity (again, example frames are shown for each stimulus type).

A common carrier was used for all stimuli presented, consisting of a dynamic, high-contrast pseudo-checkerboard varying in spatial frequency and phase (Figure 1A). Three stimulus configurations were presented, differing only in the arrangement of apertures revealing portions of the carrier pattern.

Firstly, a size-invariant bar stimulus (Figure 1B) consisting of a 2.70° bar drifting along four possible directions; horizontal, vertical and oblique diagonals. Each trial consisted of a bar sweep along a given direction, a second sweep along its orthogonal direction and a blank period of mean luminance gray background (see Supplementary Material). This was followed by a second trial for which the direction of motion was reversed. We conducted a total of two trials per run. Each sweep of the bar aperture encompassed 24 volumes and each blank period 24 volumes, totaling 144 volumes per run. Two types of runs were conducted, one with stimulus sweeps along cardinal axes (0°, 90°) and one along oblique axes (45°, 135°). We collected two runs with opposite sweep directions for each type.

The second stimulus consisted of a bar scaled in width by eccentricity (Figure 1C). Eccentricity was scaled according to the inverse natural logarithm of eccentricities between 0.06° at fixation and 9° or 16° at maximal periphery, in order to account for cortical magnification in visual cortex (Cowey and Rolls, 1974; Rovamo and Virsu, 1979). Once more, these were runs with bar sweeps along cardinal and oblique directions (see Supplementary Material). Two runs with opposite sweep directions were collected for each type, with each run totaling 144 volumes. This stimulus was implemented in a sub-set of four out of eight participants, in order to explore the effects of eccentricity scaling.

Third, a “wedge and ring” stimulus configuration defined in polar angle coordinates was presented (Figure 1D). This configuration has been previously described in the context of phase-encoded retinotopic mapping (Furuta et al., 2005), but not implemented in pRF mapping. The stimulus consisted of two simultaneously presented apertures; one triangular (“wedge”), comprising 18° of the disc circumference, rotating clockwise or counter-clockwise around fixation plus an expanding or contracting annulus (“ring”) aperture. The ring component varied with eccentricity, increasing or decreasing in radius following a logarithmic function, with 50% of overlap between adjacent aperture steps. The wedge component did not vary in size, with 50% overlap between adjacent aperture steps (see Supplementary Material). Both apertures cycled at different frequencies: 20 and 15 volumes were acquired for a single revolution of wedge (6 cycles) and rings (8 cycles), respectively. Two runs of the composite stimulus configuration were presented once in each direction of motion (clockwise/expanding and counter-clockwise/contracting) for 144 volumes each, including 24 mean luminance blank volumes in the final segment of each run. Therefore, a total of 288 volumes were collected, with a mean luminance blank period placed between the runs and a second blank period at the end of the second run.

Finally, we estimated the individual hemodynamic response function (HRF) of visual cortex for each participant. In an additional run we presented short photic bursts consisting of a full-field (radius 16° visual angle) aperture of the carrier pattern described above. A burst was presented for 1 volume and followed by 11 volumes of mean luminance blank screen. This was repeated 10 times, with a run totaling 120 volumes. Additionally, a fixation dot changing color pseudo-randomly was presented throughout, in all stimulation conditions in order to ensure participant fixation.

#### Quantitative description of stimuli

All stimulus configurations shared the same underlying high-contrast carrier pattern but differed only in the arrangement of apertures revealing portions of the pattern. The pattern was defined by the following function;

$$I(x,y)\;=\;\sqrt{x^{2}\;+\;y^{2}}\;\cos\left\{\frac{2\pi (\sin\frac{\delta \pi x}{180}\;+\;\cos\frac{\delta \pi y}{180})}{4}\;+\;\theta \right\}$$

Here *I* is the pixel intensity at **Cartesian** coordinates *x* and *y* relative to the center of the screen, and the other parameters, θ and δ, configure the phase and spatial frequency of the pattern. The θ parameter varied across time from 0 to 4π in 72 equal steps of 32 ms duration and thus completed one cycle approximately every 1.15 s. The final parameter, δ, was a function of θ, given by;

$$\delta \;=\;\frac{\sin\theta }{4}\;+\;\frac{1}{2}$$

Pixel intensities, *I*, were then rectified such that all positive values were set to maximum luminance **(white)** and all negative and zero values were set to minimum luminance **(black)**. We then bounded each resulting frame within a circular region with radius 16° by setting all pixels outside that radius to mean luminance. Within a short band (12 pixels in width) at the fringes of the patterned region we scaled the contrast of each pixel linearly with distance from the center to produce a blurred contrast edge.

The carrier therefore consisted of a dynamic, high-contrast pattern within a disc with radius 16° comprising square tessellated blocks 2.67° in diameter. Each block contained a drifting “ripple-like” pattern of concentric shapes that varied across time in spatial frequency and phase. The motion in adjacent blocks thus varied in a checkerboard-like fashion between expansion and contraction. Finally, the overall orientation of the pattern was varied across trials in the experiment (see details below). Because of the motion energy, the square-wave luminance modulation, and the varying spatial frequencies, this pattern was very broadband to ensure maximal stimulation of visually responsive neurons. The mean luminance of the stimulus was 775.05 cd/m^2^.

While the conventional checkerboard design is used commonly in the literature, its implementation here would result in unwanted differences with regard to spatial frequency information between conditions, different low-level stimulus attributes, edge artifacts and energy confounds. For example, a standard “dartboard”-type polar checkerboard provides higher spatial frequency stimulation in the central visual field compared to the periphery. In addition, using such a carrier stimulus with bar apertures results in an apparent “swaying” motion percept. Similarly, a Cartesian checkerboard displayed under bar apertures results in edge artifacts and different energy contents at various aperture positions. In order to compare bar stimuli with the simultaneous wedge and ring stimuli in a more balanced way, we implemented the broadband stimulus described above, affording greater homogeneity in spatial frequency. Virtually any stimulus content that drives visual responses may be used to sample retinotopic properties, with more complex stimuli such as natural scenes may be more effective in localizing higher visual areas (Saygin and Sereno, 2008; Huang and Sereno, 2013). Here, we have favored a checkerboard-like pattern with homogenous spatial frequency distribution across eccentricity to remain as close as possible to the standard checkerboard pattern as possible while being matched as closely as possible between the three experimental conditions.

All three stimulus configurations described above contained the carrier pattern, but presented through different aperture configurations that clipped the stimulus pattern accordingly (see Figure 1). In all cases, the apertures changed position every 2.55 s on the onset of each acquired volume. The overall orientation of the stimulus pattern was determined by the trial condition. During presentation of bars with cardinal orientations (vertical, horizontal), the pattern was not rotated. During presentation of oblique bars, the pattern was rotated 45°. During presentation of the simultaneous wedge and ring stimulus, the pattern was rotated by the same angle as that of the wedge.

The fixation dot was a blue disc of 0.42° diameter. It was surrounded by a 1° gap of mean luminance gray. Within the inner 0.5° of the mapping stimulus nearest to fixation the stimulus contrast was ramped up linearly. Each imaging run was subdivided into short time bins 200 ms in duration. During each of these bins there was a 0.05 probability that the fixation dot would change color to purple only constrained by the condition that the previous bin did not already contain a color change. Participants were instructed to maintain fixation at all times and to monitor the fixation dot for color changes upon which they were to press a button on an MRI-compatible response box.

### MRI acquisition and pre-processing

Functional MR images were acquired on a Siemens 3T Magnetom Trio using a 32-channel head coil (Siemens, Erlangen, Germany). To avoid visual field restrictions we only used the bottom element of the head coil, totaling 20 channels. A gradient-echo echo-planar imaging (EPI) sequence was used (*TR* = 2550 ms, *TE* = 37 ms, 30 interleaved slices), with off-axial acquisition and effective resolution of 2.3 × 2.3 × 2.3 mm^3^. A T1-weighted anatomical image (*TR* = 7.92 ms, *TE* = 2.48 ms, resolution = 1 × 1 × 1 mm^3^) was acquired in-plane with the functional protocol to aid registration. B_0_ maps (*TR* = 1020 ms, *TE* = 12.46 ms, resolution = 3 × 3 × 3 mm^3^) were measured to estimate local field distortions. Finally, a high-resolution T1-weighted volume (*TR* = 1900 ms, *TE* = 2.97 ms, resolution = 0.5 × 0.5 × 1 mm^3^) was acquired with the full head coil arrangement and used to reconstruct the cortical surface.

Each participant underwent one MRI scanning session, beginning with a T1-weighted anatomical image and followed by 10 runs of fMRI acquisitions; 2 runs of cardinal size-invariant bar stimulus, 2 runs of oblique size-invariant bar stimulus, 2 runs of cardinal logarithmically-scaled bar stimulus, 2 runs of oblique logarithmically-scaled bar stimulus and 2 runs of simultaneous wedge and ring stimulus. Totaling 1440 volumes acquired per participant. The order of presentation was counterbalanced between participants. Next, photic stimulation was presented for HRF estimation for 120 volumes, followed by the B_0_ map. Finally, a high-resolution T1-weighted volume was acquired. Total imaging time for each participant was approximately 80 min.

High-resolution anatomical images were processed with FreeSurfer (Dale et al., 1999; Fischl et al., 1999) for white and gray matter segmentation and cortical surface reconstruction. A manual definition of the occipital lobe surface was created for each hemisphere in order to restrict data analysis to the posterior regions of cortex. Pre-processing of functional images was carried out in SPM8 (Wellcome Trust Centre for Neuroimaging, http://www.fil.ion.ucl.ac.uk/spm). The first 4 volumes of each functional run were removed to allow for T1 equilibration effects. All images were then bias-corrected, realigned to the first image of the run and unwarped to correct for movement artifacts and field distortions (Friston et al., 1995a; Ashburner and Friston, 1997, 2005; Andersson et al., 2001; Hutton et al., 2002). We performed slice-timing correction to avoid variations in the time series due to the timing of slice acquisition. The images were subjected to a two-step registration; first to the anatomical scan acquired in-plane with the functional images and then to the high-resolution anatomical image that was acquired using the full 32-channel head-coil. Finally, the data were projected onto the reconstructed surface for each participant by interpolating volumetric data at each vertex location using a nearest-neighbor algorithm, and selecting the vertices falling at the median distance between the pial and white matter surfaces.

### Phase-encoded analysis

All subsequent data analyses were conducted using custom MATLAB (v.8.0) software. Data from the simultaneous wedge and ring stimulus configuration were analyzed with a traditional phase-encoded retinotopic mapping approach (Sereno et al., 1995; DeYoe et al., 1996; Engel et al., 1997). In brief, the mean luminance blank periods at the end of each run were removed. Smoothing was applied using a Gaussian full width at half maximum (FWHM) kernel of 5 mm on the inflated spherical surface, and data converted to relative signal change (% BOLD change) by de-trending and de-meaning individual time series. Each run was analyzed independently via a fast Fourier transform procedure at each vertex to determine the power and phase at the two fundamental frequencies (6 cycles per run for polar angle, 8 cycles for eccentricity). As a result, the signal phase at the stimulation frequency of wedges reflected the polar angle position, and the signal phase at the stimulation frequency of rings reflected the eccentricity position at each vertex. Finally, HRF lag effects were discounted by averaging phase maps across runs of the stimuli cycling in opposite directions.

### Population receptive field (pRF) estimation

**Figure 2:**
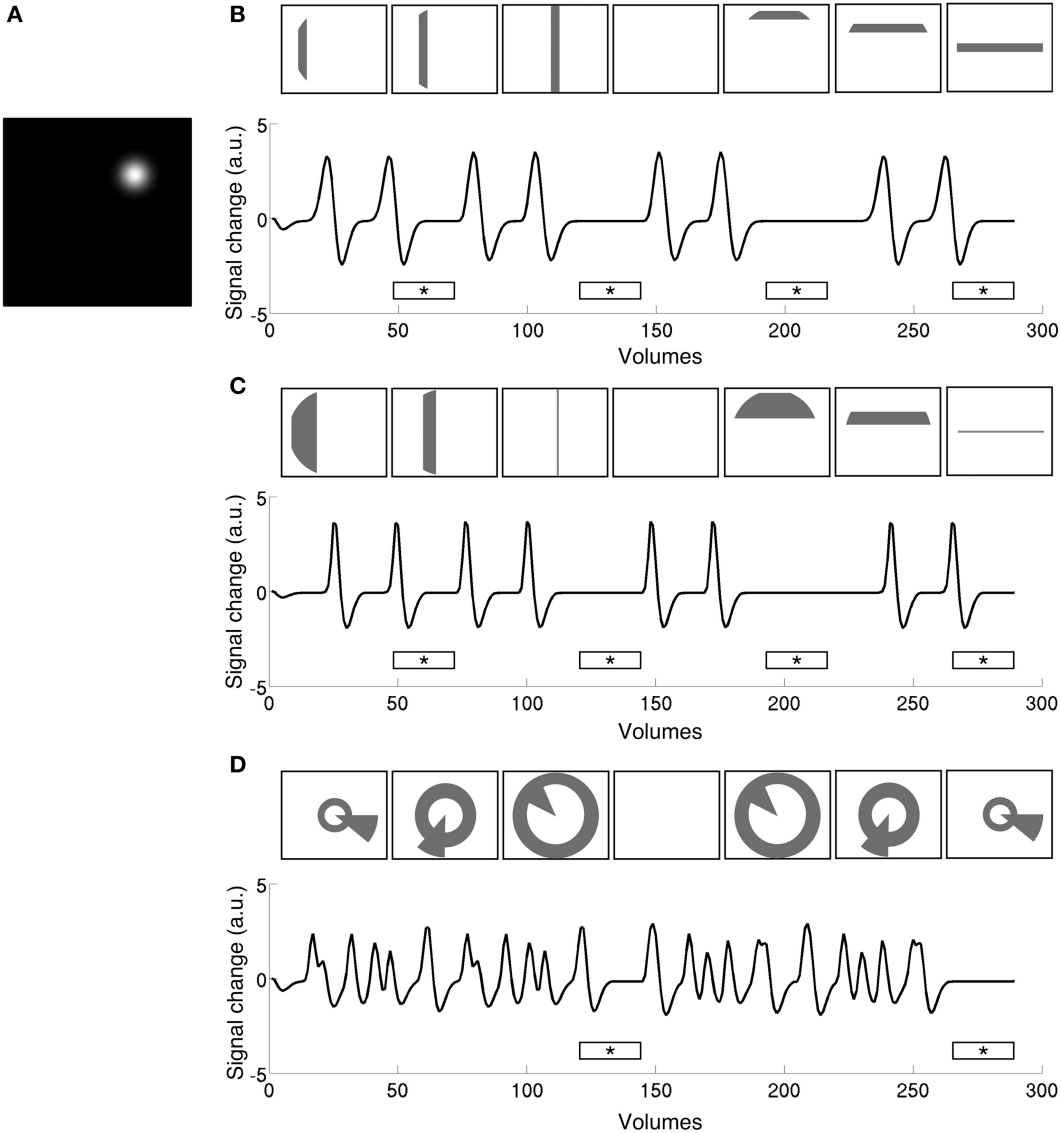
**Time series predictions for stimulation to a single pRF (A) under size-invariant bars (B), logarithmically-scaled bars (C), and simultaneous wedge and ring (D), stimulus configurations**. Use of a standard bar design produces large baseline zones that are uninformative to the model as to pRF location and spread. In contrast, the simultaneous wedge and ring stimulus based on polar angle coordinates stimulates the pRF more frequently, providing more elicited events fitted by the model. Stimulus frames are illustrative and do not correspond to specific time points along the time-series. Mean luminance periods indicated by asterisk bars.

We took a forward-modeling approach to the functional data to estimate the receptive field properties of the underlying neural populations based on Dumoulin and Wandell (2008) and as used by us in previous studies (de Haas et al., 2014; Schwarzkopf et al., 2014). The pRF model we employed is a two-dimensional Gaussian described by four parameters: two encoding the visual field position in Cartesian coordinates (X_0_, Y_0_), the spatial spread of the receptive field (σ), and the amplitude of the signal (ß). We estimated these parameters for the time series at each vertex of the sampled cortical surface, restricted to an occipital region of interest delineated manually on the inflated cortical surface, in five main steps:

1. *Model creation*. The model rests on the prior knowledge of the stimulus aperture presented for each stimulus configuration, and the assumption of a simple Gaussian receptive field. A three-dimensional search space of possible combinations of location and receptive field size was created within bounds of the maximum eccentricity stimulated (see above for details of the visual display). This search space was then sampled for candidate locations in X_0_ and Y_0_ in steps of 2.4° and σ values in 34 exponentially incremental steps from 0.32° to 32°. We created a predicted neural time series for each combination of these parameters by calculating, the sum of Gaussian receptive field weights that fall within a binary stimulus aperture for each time point of the stimulus.
2. *HRF estimation*. The HRF was estimated on a per-participant basis by taking the photic stimulation data, identifying and removing outlier values which departed more than 1.5 standard deviations from the mean for each trial, averaging the signal across trials and then fitting a double gamma function (Friston et al., 1995b). The free parameters modeled were the delay of the response, the delay of the undershoot and the ratio between these two parameters, all relative to the onset of the stimulus.
3. *Surface smoothing*. Spatial smoothing (FWHM kernel = 8.3 mm) was applied to functional data on the inflated spherical surface to reduce local minima for the model fitting and produce a local-scale conjugate, which reflected the broad response at the supra-voxel level.
4. *Coarse fit*. Time series predictions generated using the search space parameters (Figure 2A) were convolved with the HRF estimation to produce a predicted time series (Figures 2B–D). Each resulting time series was then compared to the smoothed data at a given vertex, calculating the Pearson correlation between smooth data and prediction. The parameter values of the prediction that yielded the highest correlation were used as starting parameters for the fine fit. Only positively correlated vertices with a high enough coefficient of determination, *R*^2^ > 0.05, were included in fine fitting.
5. *Fine fit*. The un-smoothed data were compared to the prediction and the parameters of the model were fitted, aiming to minimize the squared residuals between data and prediction. The Nelder-Mead algorithm for unconstrained non-linear minimization (Lagarias et al., 1998) was used (implemented as the function *fminsearch* in MATLAB v8.0) for parameter optimization, using the results of the coarse fit as starting point. In addition to pRF position and size, this step also explicitly modeled the signal amplitude (ß). The resulting parameter maps were then projected onto an inflated cortical surface for rendering.

**Figure 3:**
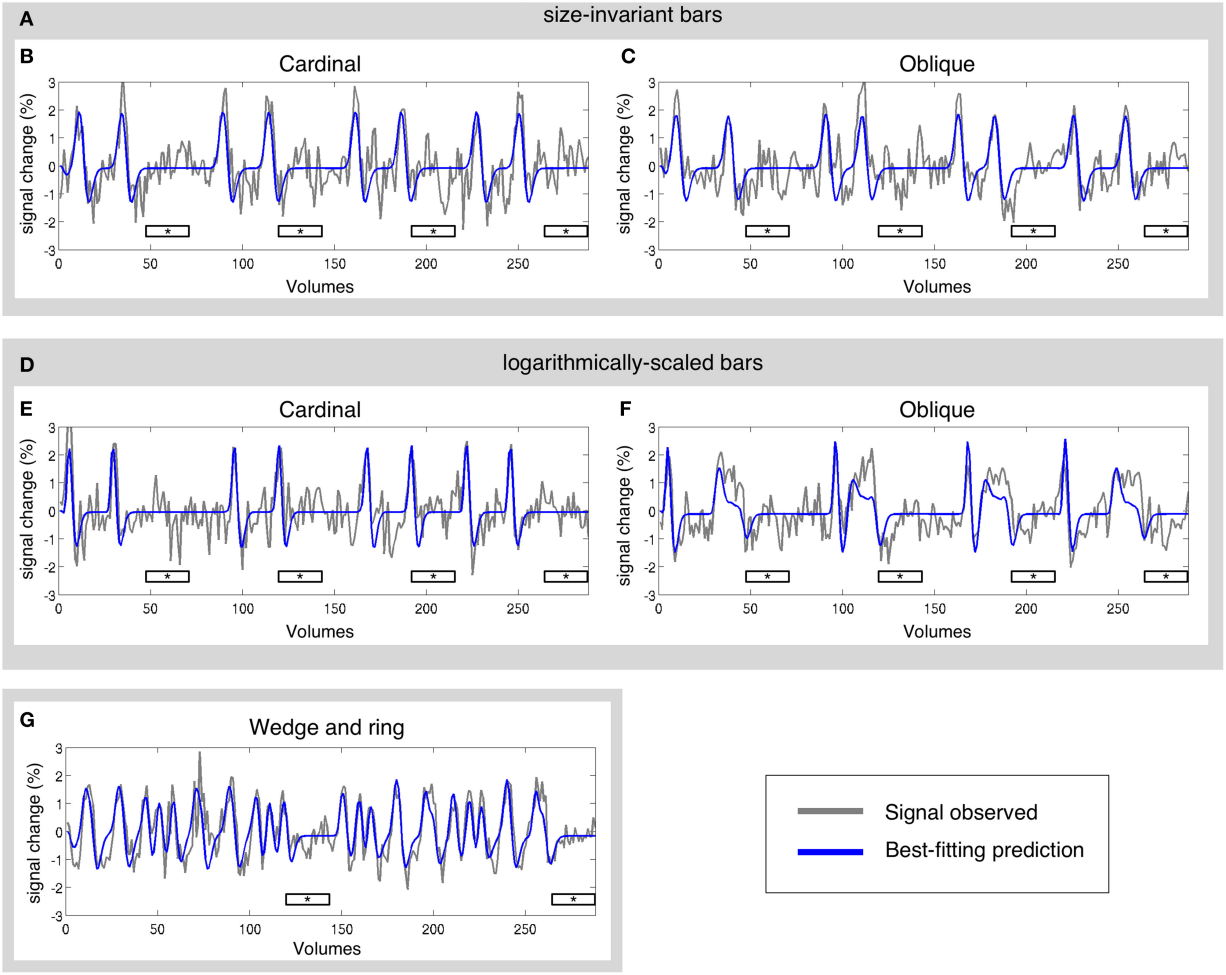
**Sample time-series and best-fitting model prediction for one vertex (cortical surface element) in area V2 from a representative participant**. Time-series are presented for three main conditions and four sub-conditions; **(A)** size-invariant bars, including its **(B)** cardinal and **(C)** oblique sweep directions; **(D)** logarithmically-scaled bars, including its **(E)** cardinal and **(F)** oblique sweep directions; and finally **(G)** simultaneous wedge and ring stimulation. Mean luminance periods indicated by asterisk bars. All conditions were fitted independently.

The pRF model estimation was performed independently for each of the three stimulus configurations (size-invariant bars, logarithmically-scaled bars, and the simultaneous wedge and ring stimulus) for each participant. Sample time-series and best-fitting model predictions under each condition are presented in Figure 3. As a further level of analysis, the size-invariant and logarithmically-scaled bar conditions were each split by run into cardinal (Figures 3B,E) and oblique directions (Figures 3C,F) and again fitted by pRF model estimation independently.

### Model cross-validation

In order to assess the performance of pRF models derived from different stimulation conditions, we performed a series of cross-validation procedures on data acquired under size-invariant and simultaneous wedge and ring stimulation. In each test, the time series observed in one stimulus condition (validation dataset) was predicted by a pRF model generated from an independent stimulus condition (training dataset) and the known stimulus aperture of the predicted condition. Independent model predictions and observed time series were then correlated to assess the performance of each model and more broadly, the generalizability of pRF models to different conditions of stimulation.

Two target datasets were selected to be predicted: cardinal and oblique directions of size-invariant bar stimulation. In turn, each target dataset was predicted by a pRF model based on (a) opposite-direction bars stimulation or (b) simultaneous wedge and ring stimulation. Correlation coefficients between model predictions and observed target time series were calculated and transformed into Fisher's *z*-values. Resulting z values were averaged across vertices of each region of interest and compared between conditions by aggregating predictions from opposite bar orientations and predictions from wedge and ring stimulation.

## Results

**Figure 4:**
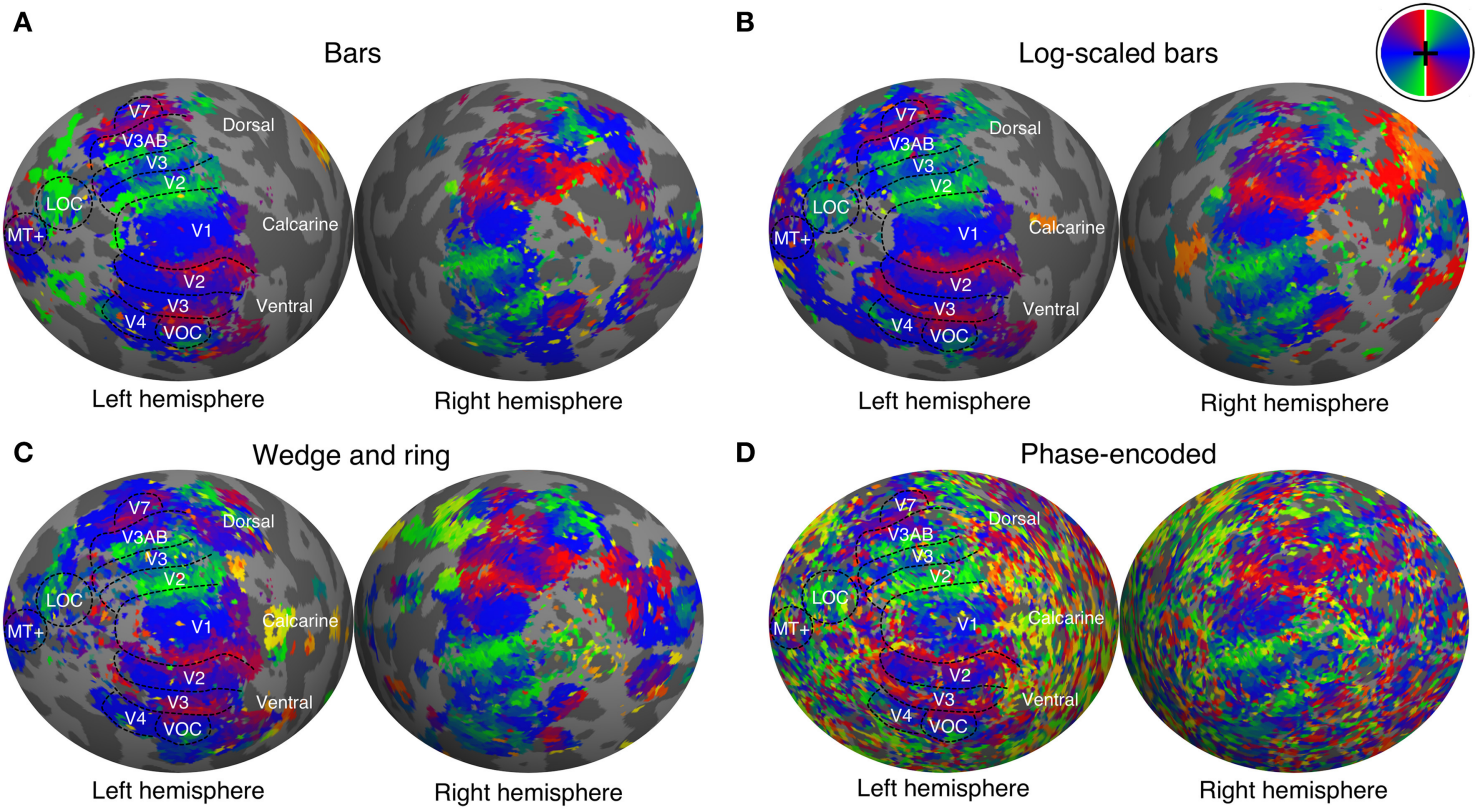
**Polar angle maps overlaid on inflated left hemisphere for a representative participant**. Polar angle estimates were derived independently from population receptive field modeling under stimulation by **(A)** size-invariant bars, **(B)** logarithmically-scaled bars and **(C)** simultaneous wedge and ring stimuli. **(D)** In addition, phase-encoded analysis of simultaneous wedge and ring stimulus is presented. Regions of interest are labeled and boundaries highlighted for the size invariant bars condition, which was used to identify those regions. Color corresponds to visual field position, as indicated by the color wheel in the upper right corner.

We manually delineated retinotopic maps in FreeSurfer using the polar angle and eccentricity representations derived from the pRF model. Early visual areas including primary visual cortex (V1), areas V2, V3, V3A, V3B, V4, V5/MT+, and V7 were reliably identified under all conditions of stimulation (see Figure 4 for a representative participant). Region delineations were performed under the size-invariant bar condition for all subjects. We did not find the defining boundary between lateral occipital areas LO-1 and LO-2 in all participants, and therefore favored a joint definition of the lateral occipital complex (LOC). Similarly, we identified a ventral occipital complex (VOC) in all participants. We were able to localize areas V1, V2, V3, V3A, V4 in data analyzed with the phase-encoded method, but localization of higher areas V5/MT+, V7, LOC, and VOC was poor. Based on visual inspections, retinotopic boundaries were better characterized by the pRF method when compared with the phase-encoded method.

### Model validation

**Figure 5:**
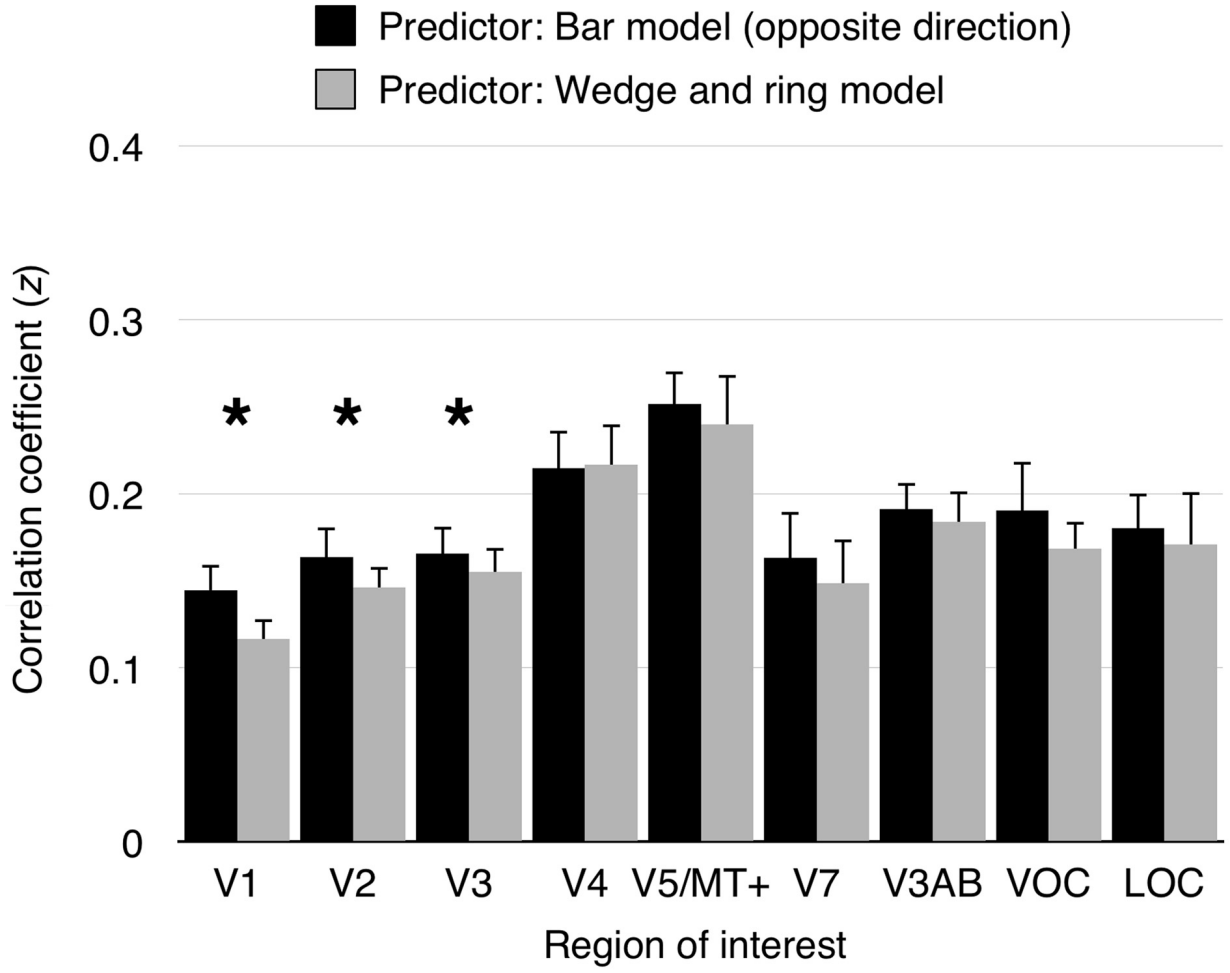
**Model cross-validation**. Cardinal and oblique orientations of size-invariant bars were predicted by a pRF model derived from either opposite bar direction or simultaneous wedge and ring stimulation. Model prediction were correlated with the signal observed in the predicted condition. Data from cardinal bars predicted from the oblique bars pRF model and oblique bars predicted from the cardinal bars pRF model were collapsed into “opposite bar direction” predictions. Similarly, predictions of either cardinal or oblique bars signal predicted from the simultaneous wedge and ring pRF model were collapsed into simultaneous wedge and ring predictions. Correlation coefficients were transformed to *z*-values (Fisher's z-transformation). A significant difference in prediction was observed in areas V1 (*z difference* = 0.03), V2 (*z difference* = 0.02), and V3 (*z difference* = 0.01), but not in higher visual areas. Asterisk indicates significant difference between conditions (*p* < 0.05).

To assess the performance of pRF models derived from different stimuli, we performed a series of cross-validation tests. First, pRF models were estimated from data obtained under cardinal and oblique bar stimulation independently, as well as from simultaneous wedge and ring stimulation. Second, predictions were generated from two conditions; either cardinal or oblique bar pRF models and simultaneous wedge and ring pRF models. Finally, the model predictions were compared against an independent *observed* time series, obtained under the remainder condition. The bar pRF model outperformed the wedge and ring pRF model in prediction accuracy in areas V1 (*t* = 5.55, *df* = 15, *p* < 0.001), V2 (*t* = 3.47, *df* = 15, *p* < 0.01), and V3 (*t* = 2.64, *df* = 15, *p* < 0.05), but not in any other region of interest (see Figure 5). This reliability advantage is not surprising, considering the geometric similarity between cardinal and oblique sweeping bars designs. Nevertheless, the difference in reliability was extremely small, with a maximum predictive difference of *z* = 0.03 (*R^2^* < 0.001) between bar and wedge and ring predictor models.

### Goodness of fit

Having demonstrated comparable reliability across the different experimental conditions tested, we compared goodness of fit as quantified by the coefficient of determination (R^2^) of our experimental conditions. Strictly, a direct comparison of the conditions based solely on the goodness of fit would be misleading because they are not only based on different models but also on different data. As such it would be incorrect to interpret differences in goodness of fit as differences in efficiency or reliability of the procedure. However, in practical terms, the goodness of fit achieved by each procedure is important because, provided precision of each method is similar, the goodness of fit determines how many voxels survive statistical thresholding, i.e., the statistical power of the method. Furthermore, the models compared differed only in visual stimulation and not in the number of free parameters or the nature of the model fitted.

We compared three stimulus configurations: size-invariant bars, bars varying logarithmically in size with eccentricity, and a simultaneous wedge and ring aperture, also scaled logarithmically for eccentricity. In addition, we also included independent pRF fits for the cardinal and oblique directions of both size-invariant and logarithmic bars, as sub-conditions. To ensure parameter estimates were comparable, we thresholded each vertex within a region of interest at goodness of fit values *R*^2^ > 0.1. The number of vertices surviving thresholding did not differ significantly across conditions (ANOVA, *F* = 0.48, *df* = 3, *p* = 0.726).

**Figure 6:**
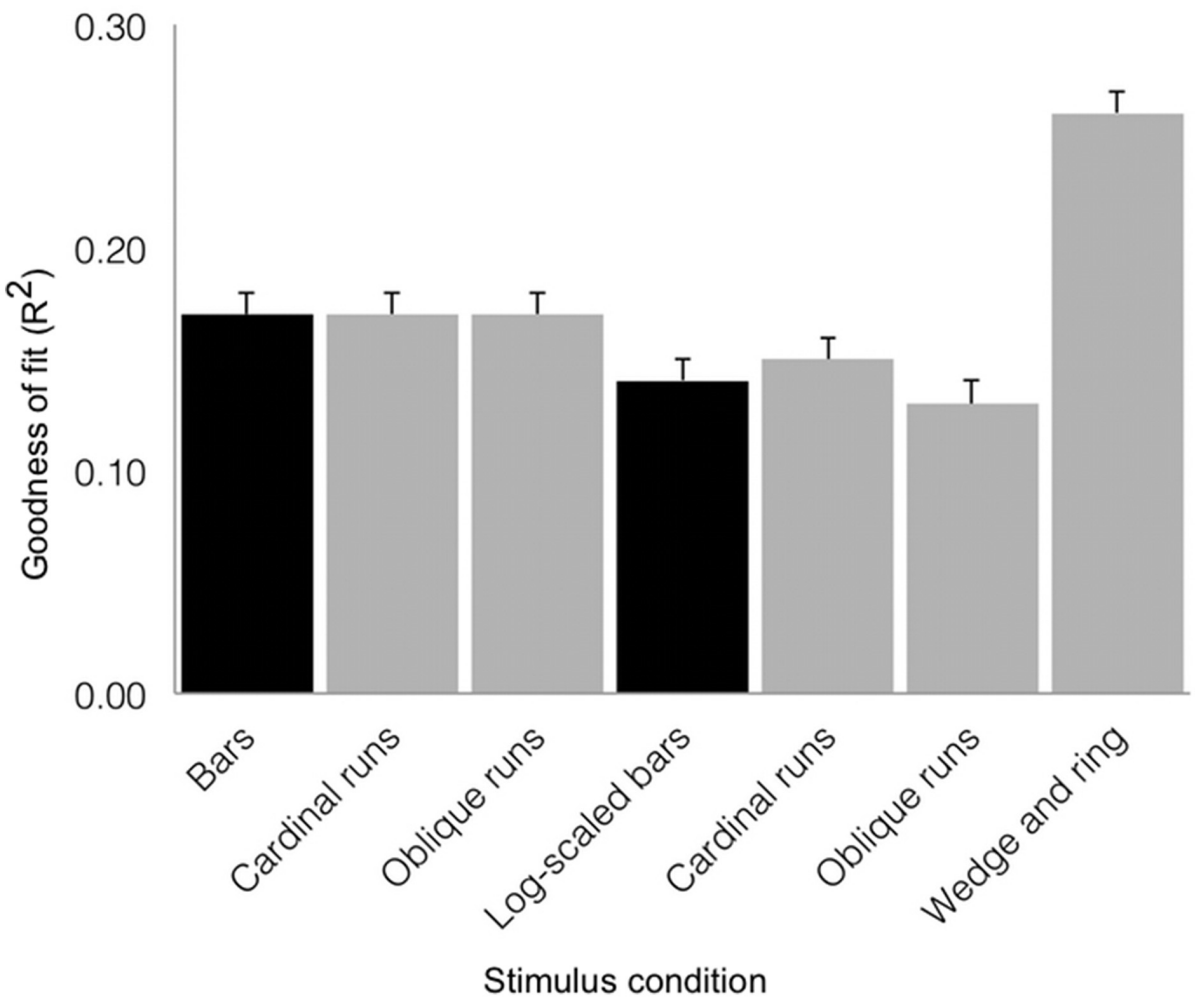
**Group average goodness of fit (R^2^) for the three stimulus configurations as derived from sum of squared residuals between the observed time series and model predictions for regions V1, V2, and V3 combined (error bars correspond to standard errors of the mean)**. Black shading denotes estimates from 576 volumes acquired and gray shading denotes estimates derived from 288 volumes acquired. Simultaneous wedge and ring stimulation produced better data fits, while requiring half the data compared to size-invariant or logarithmically-scaled bars collapsed across bar directions.

We tested goodness of fit (R^2^) values across stimulus conditions with a repeated-measures design ANOVA (see Figure 6). We found a main effect of condition (*F* = 29.37, *df* = 6, *p* < 0.001), with no significant contribution of inter-subject variability (*F* = 1.48, *df* = 18, *p = 0.101*). The simultaneous wedge and ring stimulus configuration afforded marginally better fits (*mean R*^2^ = 0.26 ± 0.01 *SEM*) than either the size-invariant (*mean R*^2^ = 0.17 ± 0.01 *SEM*) or logarithmically-scaled (*mean R*^2^ = 0.14 ± 0.01 *SEM*) bar stimuli. Crucially, these results rested on only half the amount of data and scanning time compared to size-invariant or logarithmically-scaled bars, indicating a time-to-acquire advantage.

### Effect of mean luminance periods on pRF estimates

To accurately estimate pRF parameters, a baseline measure is introduced by acquiring data during a mean luminance period while no modulation of contrast or spatial frequency is taking place (Dumoulin and Wandell, 2008). The bar stimuli and the simultaneous wedge and ring stimulus conditions in our design were matched for the number of data points, that is, each scanning run comprised 144 volumes regardless of condition. This means that both runs of the bar stimulus with opposite directions and the two directions of the simultaneous wedge and ring stimulus each totaled 288 volumes. Therefore, the benefit in goodness of fit for the wedge and ring stimulus could not trivially be explained by the amount of data collected in each condition—in fact, it remained even when twice the amount of data was used for the bar stimuli by collating runs with cardinal and oblique sweep directions.

However, it could be argued that the length of mean luminance periods affects the goodness of fit. Compared to the simultaneous wedge and ring design, in the bar design there were twice as many volumes during which mean luminance frames were presented. As Figure 2 shows, the reduced goodness of fit for the bar stimuli could therefore be due to the fact that there was more unexplained variance caused by the longer periods during which the model predicted a zero response. In order to test this possibility, we performed a control analysis where the number of mean luminance volumes acquired during size-invariant bar stimulation was truncated from 48 to 24 volumes per run to match the number of mean luminance volumes in a single run of the simultaneous wedge and ring stimulus. The resulting truncated data were then fitted with the pRF model described above, revealing a significant difference between the original (*mean R*^2^ = 0.14 ± 0.01 *SEM*) and truncated (*mean R*^2^ = 0.17 ± 0.01 *SEM*) models (*t* = 3.85, *df* = 7, *p* < 0.01). However, this difference was markedly smaller than the advantage afforded by the ridge configuration, and within the variability range of the stimuli probed here, indicating the number of mean luminance volumes did not fully account for goodness of fit differences observed between bar-type and wedge and ring stimuli. Moreover, this modest increase in the goodness of fit after truncation was accompanied by a decrease in the degrees of freedom.

### Stimulus configuration and pRF size

The size of the pRF denotes the two-dimensional spread of the visual field locations from which responses in the sampled vertex can be elicited and is quantified by the standard deviation (σ) of the two-dimensional Gaussian in degrees of visual angle. Typically, the size of single neuron receptive fields increases with eccentricity (Hubel and Wiesel, 1977; Van Essen et al., 1984) and along the visual map hierarchy in primates (Zeki, 1978; Maunsell and Newsome, 1987; Felleman and Van Essen, 1991). Similarly, pRF sizes also scale with eccentricity and position in the visual hierarchy (e.g., Dumoulin and Wandell, 2008). Here, we tested the effects of our three different stimulus configurations on pRF size estimates.

#### pRF size: size-invariant vs. logarithmically-scaled bar apertures

**Figure 7:**
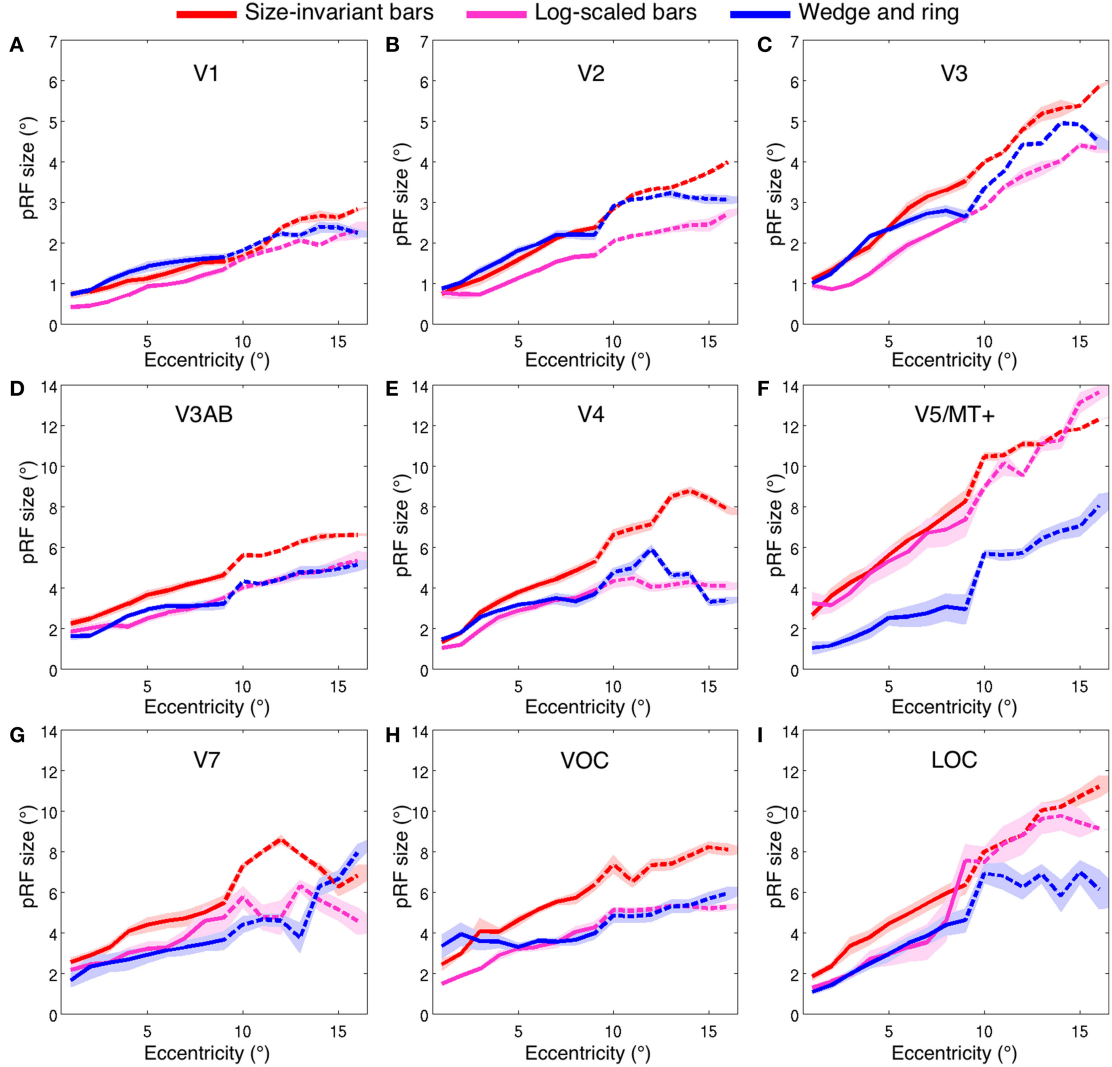
**Population receptive field size (σ, in degrees of visual angle) across two ranges of visual field eccentricities; 9° (solid line, six participants) and 16° (dashed line, two participants)**. Vertices were binned and averaged across participants in steps of 1° of eccentricity and plotted for three stimulus configurations; size-invariant bars (red), logarithmically-scaled bars (magenta) and simultaneous wedge and ring stimulus (blue). Nine bilateral regions of interest displayed; **(A)** V1, **(B)** V2, **(C)** V3, **(D)** V3AB, **(E)** V4, **(F)** V5/MT+, **(G)** V7, **(H)** VOC and **(I)** LOC. Shaded area corresponds to standard error of the mean.

Estimates for V1, V2, and V3 followed the expected pattern of monotonic increase with eccentricity (Figures 7A–C). pRF sizes estimated from the size-invariant bar conditions covered approximately 0.7–2.8° in V1, 0.8–4.0° in V2 and 1.1–5.8° in V3, in broad agreement with values reported in the literature (Smith et al., 2001; Dumoulin and Wandell, 2008; Amano et al., 2009; de Haas et al., 2014; Schwarzkopf et al., 2014). These results were in contrast with bar apertures accounting for the effects of cortical magnification, i.e., expanding logarithmically with eccentricity, as tested in a subset of 4 participants. The latter provided significantly lower pRF size estimates for V1 (0.4–2.3°), V2 (0.7–2.7°) and V3 (0.8–4.4°) (all participants *p* < 0.001, see Table 1 for pairwise *t*-tests).

**Table 1:** **Within-subject pairwise *t*-tests assessing differences in pRF size (in degrees of visual angle) between (a) size-invariant and logarithmically-scaled bars and (b) size-invariant bars and simultaneous wedge and ring stimulus design across nine regions of interest**.

**Participant**	**V1**	**V2**	**V3**	**V4**	**V5**	**V7**	**V3AB**	**VO**	**LO**
**SIZE INVARIANT BAR vs. LOGARITHMICALLY SCALED BAR STIMULATION**									
1	3.67 (3682)^***^	41.25 (2463)^***^	32.76 (1921)^***^	48.59 (1157)^***^	39.07 (509)^***^	17.56 (649)^***^	34.94 (952)^***^	45.62 (673)^***^	28.85 (1351)^***^
2	65.35 (2239)^***^	52.72 (1416)^***^	53.16 (1256)^***^	70.59 (954)^***^	21.92 (327)^***^	42.91 (593)^***^	63.16 (776)^***^	45.60 (136)^***^	69.21 (1883)^***^
3	19.08 (3697)^***^	7.90 (2872)^***^	30.88 (2655)^***^	49.66 (1616)^***^	31.77 (537)^***^	25.88 (664)^***^	39.68 (1721)^***^	31.96 (374)^***^	15.66 (1314)^***^
4	23.49 (2918)^***^	30.63 (2330)^***^	36.33 (1981)^***^	30.77 (1201)^***^	22.87 (471)^***^	5.84 (223)^***^	53.41 (1150)^***^	10.86 (212)^***^	10.73 (630)^***^
5									
6	No logarithmically-scaled bar stimulation								
7									
8									
**SIZE INVARIANT BAR vs. SIMULTANEOUS WEDGE AND RING STIMULATION**									
1	0.73 (3834)^**^	0.51 (2501)^**^	25.29 (1922)^***^	72.09 (1203)^***^	28.34 (384)^***^	0.35 (525)^*^	34.18 (1147)^***^	30.65 (464)^***^	19.98 (1609)^***^
2	3.00 (2378)^***^	8.67 (1646)^***^	36.07 (1339)^***^	50.34 (1173)^***^	32.29 (546)^***^	25.53 (730)^***^	36.20 (1037)^***^	13.86 (70)^***^	36.34 (2257)^***^
3	15.72 (4061)^***^	17.07 (2868)^***^	5.87 (2501)^***^	30.42 (1506)^***^	46.66 (399)^***^	36.46 (593)^***^	38.27 (1831)^***^	31.20 (439)^***^	24.41 (1226)^***^
4	50.58 (3253)^***^	15.47 (2288)^***^	0.98 (1842)^**^	9.80 (1129)^***^	6.82 (201)^***^	15.15 (286)^***^	20.85 (1342)^***^	6.10 (249)^***^	1.53 (361)^**^
5	13.45 (2564)^***^	7.22 (1951)^***^	18.90 (1462)^***^	12.09 (434)^***^	18.72 (2)^***^	3.37 (31)^***^	12.32 (390)^***^	0.62 (90)^**^	9.03 (77)^***^
6	13.84 (2862)^***^	4.40 (2646)^***^	18.40 (2206)^***^	45.82 (1327)^***^	32.01 (704)^***^	15.62 (447)^***^	19.37 (1371)^***^	8.71 (472)^***^	23.93 (771)^***^
7	27.17 (3018)^***^	11.42 (2360)^***^	7.55 (1938)^***^	56.72 (1219)^***^	17.42 (95)^***^	8.75 (473)^***^	73.92 (2634)^***^	18.18 (307)^***^	24.77 (515)^***^
8	13.86 (2070)^***^	28.48 (1861)^***^	48.93 (1571)^***^	45.19 (720)^***^	–	14.56 (49)^***^	39.73 (993)^***^	26.68 (161)^***^	14.67 (72)^***^

*Positive t-values indicate larger pRFs in the first condition*.

* p < 0.05,

** p < 0.01,

*** *p < 0.001*.

We also examined the effect of stimulus configuration on pRF size in higher visual regions (Figures 7D–I). As expected from the organization of cortical visual regions, there was a monotonic relationship between hierarchical position and pRF size. Note the differentiation in pRF size estimates in ventral region V4 (Figure 7E), where the size-invariant bar conditions produced larger pRFs at highly eccentric representations. This was statistically significant when compared to the logarithmically-scaled condition (see Table 1 for pairwise *t*-tests). Results presented here for size-invariant bars are consistent with reports in the literature of pRF sizes in the range of 2–6° for area V4 (Winawer et al., 2010).

Similar effects of stimulus configuration were observed in the ventral occipital complex (VOC), encompassing map VO-1 and VO-2 and the lateral occipital complex (LOC), encompassing LO-1 and LO-2 (Figures 7H,I). Estimates of pRF size were consistent with expectations of linear increase with cortical hierarchy, with estimates for LO regions in the range of 2–9°. These data are consistent with previous estimates of pRF size for human LO regions (Larsson and Heeger, 2006; Amano et al., 2009). As a general trend, larger pRF size estimates were observed in the size-invariant bars condition compared to the cortical magnification-scaled conditions, with significant divergence between logarithmic and size-invariant bars in VOC and LOC.

#### pRF size: size-invariant vs. simultaneous wedge and ring apertures

The hierarchical increase in pRF size with cortical area across V1, V2, V3, and V4 was replicated, with smaller pRF size estimates in the simultaneous wedge and ring stimulus condition (V1 = 0.7–2.4°; V2 = 0.9–3.2°; V3 = 1.0–4.9°; V4 = 1.4–5.9°) when compared to the size-invariant condition (all comparisons *p* < 0.05, see Table 1 for individual pairwise *t*-tests). In early visual areas V1, V2, and V3 (Figures 7A–C) pRF sizes differences between conditions are non-distinguishable in central visual field representations, below 2° eccentricity. More eccentric representations, and those of higher visual areas diverge, with smaller pRF size estimates for the simultaneous wedge and ring stimulus.

Of particular interest was area V5/MT+ (Figure 7F), where the simultaneous wedge and ring condition produced significantly smaller pRFs (σ = 1.0–8.0°) than the size-invariant condition (σ = 2.7–12.3°). This is at odds with previous human fMRI data, which estimated the pRF size of V5/MT+ to be between 5° and 11° (Amano et al., 2009). The present data suggest that pRF size estimates in the region V5/MT+ may be more susceptible to stimulus configuration, compared with early visual areas.

In addition to the linear effects on pRF size, we also observed an interaction of eccentricity with pRF size differentiation across conditions. In areas V7, VOC, and LOC (Figures 7G–I), but also earlier areas in the visual hierarchy such as V4 and V5/MT+, the difference in pRF size across conditions scaled with eccentricity, with larger differences at the more eccentric positions. In order to test this, we performed a linear regression on the pRF size increment with eccentricity and compared the slope of the fits between size-invariant bars and simultaneous wedge and ring presentations across participants. For region V1, we found no difference in the linear fit slopes between conditions (*mean slope difference* = 0.01 ± 0.02 *SEM*, *t* = 0.29, *df* = 7, *p* = 0.780), while regions V2 (*mean slope difference* = 0.04 ± 0.02 *SEM*), V3 (*mean slope difference* = 0.09 ± 0.02 *SEM*), V4 (*mean slope difference* = 0.16 ± 0.05 *SEM*), V5/MT+ (*mean slope difference* = 0.34 ± 0.08 *SEM*), V3AB (*mean slope difference* = 0.10 ± 0.02 *SEM*), and VOC (*mean slope difference* = 0.40 ± 0.13 *SEM*) showed a significant slope difference between conditions (all comparisons *p* < 0.05). This indicated the wedge and ring condition consistently returned smaller pRF sizes relative to the size-invariant condition at highly eccentric representations. No significant slope differences were observed in regions V7 (*mean slope difference =* 0.23 ± 0.09 *SEM*, *t* = 2.31, *df* = 7, *p* = 0.054) or LOC (*mean slope difference* = 0.15 ± 0.10 *SEM*, *t* = 1.56, *df* = 7, *p* = 0.163).

### Effects of display size on pRF estimates

An effective stimulus design for pRF mapping is one that produces accurate estimates of the property sampled independently of extraneous factors such as viewing distance; therefore a design that introduces biases at different viewing distances is likely to be suboptimal. In order to test this, two participants viewed the stimuli described here with an eccentric coverage of 16° from fixation, while a further six participants viewed them with 9° coverage from fixation. We compared the goodness of fit (R^2^) of the pRF model in three stimulus designs (size-invariant bars, logarithmically-scaled bars and simultaneous wedge and ring stimulus) across the two viewing distances with a between-subjects univariate ANOVA model across all regions of interest. *R*^2^-values were significantly larger at 16° eccentricity compared to 9° eccentricity under the size-invariant (*F* = 8.31, *df* = 34, *p* < 0.01) and logarithmically-scaled bar stimuli (*F* = 11.14, *df* = 34, *p* < 0.01). This is unsurprising, given the larger display elicited activations in the more peripheral representations of visual space, therefore providing stimulus-related signals in a larger expanse of cortical territory, compared to the smaller display. Crucially, when comparing the goodness of fit in the wedge and ring condition, there was no significant difference between subjects who viewed the small and large screen display (*F* = 2.07, *df* = 34, *p* = 0.159), pointing to a robust fit of the pRF model in the simultaneous wedge and ring condition independent of viewing distance.

We also tested whether the model was biased in its estimates of pRF size by an interaction between display size and stimulus configuration. To assess this, we calculated the mean difference in pRF size between subjects who experienced the 9° display vs. the 16° display in 20 equally-spaced eccentricity bins, spanning the range of 0.5–9° of eccentricity for all regions of interest. We found the 16° display produced marginally larger pRFs under the size-invariant bar stimulus (*mean difference* = 0.72°), compared to the simultaneous wedge and ring stimulus (*mean difference* = 0.60°, *t* = 2.34, *df* = 8, *p* < 0.05), with both results being within the levels of observed inter-individual variability.

### pRF vs. phase-encoded estimation of stimulus eccentricity

A currently unsolved issue in modeling cortical pRF characteristics is the possible introduction of model-dependent biases. Dumoulin and Wandell (2008) argue for a bias in eccentricity estimation using phase-encoded methods, with an over-estimation of eccentricity at the lower boundaries, which is enhanced for areas with large pRF sizes.

**Figure 8:**
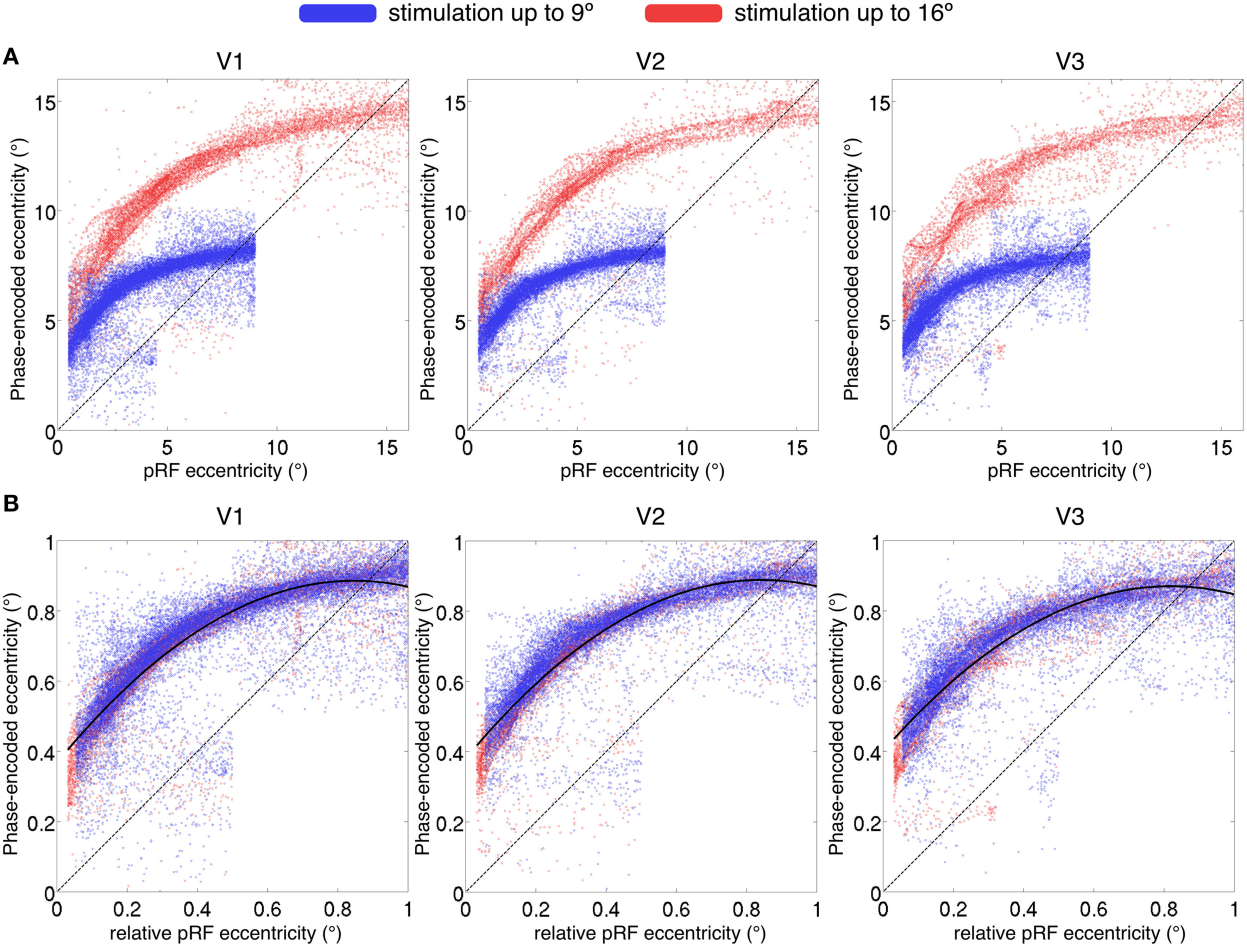
**(A)** Comparison of eccentricity estimates by pRF and phase-encoded methods for regions V1, V2, and V3. Each point represents a single surface vertex in a single participant, only vertices with goodness of fit *R*^2^ > 0.05 displayed. **(A)** The maximum eccentricity of the stimulus differed between participants (16° display in red and 9° display in blue), reproduced in the eccentricity estimates where two distinct populations are seen. **(B)** Values normalized by maximum eccentricity show a similar discrepancy for phase-encoded estimates relative to the pRF estimates, independent of maximum stimulus size. Dashed line denotes identity; i.e., a perfect correspondence between the pRF and phase-encoded estimates of eccentricity. Solid line in **(B)** denotes best-fitting second-level polynomial over all subjects.

We compared phase-encoded estimates of eccentricity, extracted from data acquired during simultaneous wedge and ring aperture stimulation, with eccentricity estimates obtained from the pRF model fitting of the same data in regions V1, V2, and V3. If there were an exact correspondence between the two eccentricity estimates, data would lie on a straight line across the eccentricity space. Instead, we found an over-estimation of the stimulus eccentricity from the phase-encoded method relative to the pRF estimates in a non-linear, eccentricity-dependent fashion (see Figure 8A). These results replicate previous findings of an overestimation of eccentricity by the phase-encoded method (Dumoulin and Wandell, 2008). Discrepancies in phase-encoded eccentricity estimates were also independent of the range of values sampled, as normalization of eccentricity values revealed similar profiles for participants presented with maximum eccentricities of 16° and 9° (Figure 8B).

## Discussion

Optimal stimulus design is an important consideration in fMRI experimental planning, both in providing targeted elicitation of the desired signals and reducing potential confounds such as physiological noise and participant motion. Here, we considered three stimulus configurations and their effects on model estimates of pRFs. We identified two factors that significantly influenced model estimates: eccentricity scaling and Cartesian vs. polar coordinate based apertures.

Three experimental conditions were compared; size-invariant bars, logarithmically-scaled bars and a simultaneous wedge and ring stimulus, with all producing comparable retinotopic maps (see Figure 4). Goodness of fit metrics revealed that conditions performed similarly, with a marginal advantage for the wedge and ring condition, even when compared to twice as much data fitted in the bar conditions (Figure 2). This advantage is important in terms of acquisition time, as prolonged scan sessions typically lead to increased subject motion, and a stimulus configuration that affords efficient estimation in a short period of time is desirable for studies where scan time is limited or the population of interest does not tolerate extended scan sessions. The advantage may be accounted for by the use of stimuli defined in polar rather than Cartesian coordinates, as within the same time period any given pRF can be stimulated more often under the simultaneous wedge and ring configuration, with more signal fluctuations recorded and accounted for by the pRF model, compared to the standard bar configurations. This effect is illustrated in Figure 2, where the pRF prediction under bar-type stimulation contains a larger proportion of uninformative periods compared to the simultaneous wedge and ring stimulus. Indeed, this advantage in more frequent elicitation of desired signals may be achieved with any stimulus configuration incorporating multiple apertures of any geometry, as long as the fundamental frequencies of apertures are de-correlated. Pragmatically, a multifocal approach with maximum length sequence (M-sequence) maximizes the efficient elicitation of signals in the shortest possible time (Buracas and Boynton, 2002; Vanni et al., 2005; Henriksson et al., 2012). While it may appear as if multifocal M-sequence approaches are therefore optimal for retinotopic stimulation, they suffer from reduced explanatory power (Ma et al., 2013). In addition, multifocal stimuli produce poorer retinotopic maps and reduced goodness of fit in pRF modeling (Binda et al., 2013), revealing a tradeoff between model accuracy and predictive power. This difference with slow traveling wave designs, such as both our bar and simultaneous wedge and ring designs, might be due to the fact that these slower designs maximize the difference between high temporal frequency noise and the mapping signal. To achieve comparable signal-to-noise ratios, a multifocal design presumably would require long epochs for each multifocal stimulus frame, which would greatly inflate data acquisition times. We therefore propose the traveling wave simultaneous wedge and ring stimulus as a potential compromise between these two competing interests in pRF mapping.

A further consideration for visual field sampling efficiency, is the heterogeneous sampling afforded by eccentricity-scaled stimuli defined in a Cartesian coordinate system, in this case, logarithmically-scaled bars. For pRFs lying outside diagonal lines in visual space, these will be sampled at different fineness, that is, the thickness of the sweeping bar, in each orientation of the sweep. As such, a pRF may be under- or over-estimated by unequal levels of fineness in each sampling orientations. This heterogeneity is not present in polar coordinate defined stimulus, such as the simultaneous wedge and ring stimulus, therefore increasing confidence in the robustness of visual space sampling.

An efficient stimulus configuration for pRF mapping not only elicits the desired signals, but also results in reliable model estimates of the underlying neuronal properties of interest. In order to assess reliability, we conducted a series of cross-validation tests. While the conventionally used size-invariant bar stimulus showed a subtle benefit in terms of reliability in early visual areas, cross-model reliability was largely similar across the conditions tested, indicating models derived from different stimulus configuration were comparable.

Nevertheless, differences in model predictions were observed, particularly in estimates of pRF size (see Figure 7). The manipulation of eccentricity scaling played an important role in pRF size estimation; size-invariant bars returned in pRF sizes in multiple cortical locations comparable to previous studies (Larsson and Heeger, 2006; Dumoulin and Wandell, 2008; Amano et al., 2009; Winawer et al., 2010), while eccentricity-scaled stimuli resulted in smaller pRF size predictions (c.f., Binda et al., 2013). In particular, the difference in pRF size was more marked for highly eccentric representations when compared to logarithmically-scaled bars and simultaneous wedge and ring stimuli. When examining extrastriate regions higher in the visual system hierarchy than early retinotopic cortices a similar pattern was found; here the effects were more marked, as these regions tend to contain voxels with larger pRFs. This was particularly true for areas such as V5/MT+ that may be more susceptible to biases by stimulus configuration compared to striate cortex. Differences in pRF size estimation may be due to the relatively large portion of the visual field covered by the logarithmically-scaled apertures, where larger field coverage at the periphery stimulates the larger receptive fields more effectively. The large field coverage design is intended to more closely reflect the known distribution of receptive field sizes in cortex; by matching net stimulus coverage with the total cortical extent, it could be argued such a design provides a more accurate estimation of receptive field properties, by incorporating the known constrains of cortical magnification. A further consideration is the possible mediation of pRF size estimates by non-classical receptive fields exerting suppressive effects. In the case of the size-invariant stimulus the suppressive effect will be minimized, as the transient bar is likely to cover a limited fraction of the non-classical receptive fields in more eccentric locations and therefore return a larger pRF size estimate. In contrast, stimuli scaled for cortical magnification have increased coverage of the non-classical receptive fields, therefore potentially increasing the contribution of surround suppression and effectively reducing the pRF size estimate. Such effects only become apparent at highly eccentric representations, where suppressive contributions are spatially differentiated by the two stimuli configurations. Previous work extended the pRF model to incorporate inhibitory surround interactions rather than merely describing it by a two-dimensional Gaussian as in the present experiments (Zuiderbaan et al., 2012; Schwarzkopf et al., 2014). By accounting for the extent and strength of inhibitory interactions, the differences in pRF sizes we observed in the different stimulus conditions could be reduced. However, in practice the center-surround pRF model does not typically differ substantially from the standard two-dimensional Gaussian model (e.g., Schwarzkopf et al., 2014).

An additional consideration regarding the influence of stimulus design on pRF size estimates, is the presence of mean luminance blank periods during stimulation. These periods allow the estimation of baseline activity at a given pRF under visual stimulation, without modulation by contrast and spatial frequency. This is particularly relevant for regions with large receptive field sizes, where baseline activity may not be easily estimated unless the stimulus aperture is removed (Dumoulin and Wandell, 2008). Arguably, modulation of the duration and temporal position of mean luminance periods may affect the pRF model estimates, including receptive field position and size. Inadequate sampling of mean luminance periods in a given stimulus design would lead to overestimation of the baseline response of large pRFs and consequently, under-estimation of pRF size. To test this, we performed a control analysis where the number of mean luminance volumes was modulated, and compared model estimates derived. This analysis revealed only very small differences in goodness of fit, emphasizing the differences in pRF estimates observed between experimental conditions were not trivially explained by the length or temporal positioning of mean luminance periods.

PRF modeling as implemented here and in previous literature relies on the assumption that a linear spatial summation of discrete components of a receptive field form an accurate picture of the whole receptive field. Recent work has shown non-linear spatial summation effects in striate and extrastriate cortex (Kay et al., 2013). The violation of such assumptions likely plays a role in the estimation of pRF size, as the spatial pattern of different stimuli conditions present different spatial integration problems, which may interact with non-linear summation effects, particularly over large receptive field locations in extrastriate regions. In addition, long-range suppression effects from distant spatial representation may play a further role in modulating estimated pRF sizes (Nurminen et al., 2010). Again, the significantly larger coverage of logarithmically-scaled or simultaneous wedge and ring stimulation is likely to lead to increased long-range suppression, and therefore reduced pRF size estimates. Whether the estimates derived from size-invariant or cortical magnification-scaled stimuli are a more veridical reflection of the underlying neuronal receptive field, remains unclear.

Finally, we found a discrepancy between eccentricity estimates from phase-encoded models compared to those based on pRF modeling of the same data, especially in more central locations. While it is possible that the particular pRF model used here introduces other biases, these results are in agreement with the previous literature (Dumoulin and Wandell, 2008) suggesting that phase-encoded methods consistently misestimate pRF eccentricity representation. Phase-encoded methods infer the pRF center location from the peak of the signal; however, particularly for ring stimuli, the maximal response may not occur when the stimulus passes the pRF center, thus resulting in poor estimation of positions in near-foveal representations. While the ground truth of eccentricity must be determined empirically, one indication that this discrepancy indeed reflects a bias in phase-encoded methods is that it is a function of relative, not absolute eccentricity (i.e., it depends on where a given pRF falls relative to the maximum eccentricity of the mapping stimulus, regardless of its absolute eccentricity: Figure 8B). Therefore model-based approaches are likely to be superior to phase-encoded analysis for the estimation of visual field position.

In summary, we have demonstrated the effects of stimulus configuration on model-based pRF estimates and identified two stimulus design factors influencing model estimates. Accounting for cortical magnification played a significant role in the estimation of pRF size, with eccentricity-scaled stimuli returning smaller pRF sizes, particularly in eccentric locations and regions with known large receptive fields (e.g., V5/MT+). Choice of Cartesian or polar coordinate-based stimuli influenced both model accuracy and predictive power, with the bar stimulus providing higher accuracy and lower predictive power, while the simultaneous wedge and ring stimulus afforded higher power, with a reduction in accuracy. Here, we demonstrate that a novel simultaneous wedge and ring stimulus provides robust model fits in a significantly reduced acquisition time, while providing comparable parameter estimates in early visual cortex, and smaller pRF size estimates in higher visual areas when compared to previously reported stimulus configurations.

### Conflict of interest statement

The authors declare that the research was conducted in the absence of any commercial or financial relationships that could be construed as a potential conflict of interest.
