# Supplementary material for: Comparing different stimulus configurations for population receptive field mapping in human fMRI
Source: Front Hum Neurosci. 2015 Feb 20;9:96. doi: 10.3389/fnhum.2015.00096 (PMC4335485; doi:10.3389/fnhum.2015.00096)
Supplement: Supplementary file 4 [file DataSheet1.DOCX]

***Supplementary Material***

**Comparing different stimulus configurations for population receptive field mapping in human fMRI**

**Ivan Alvarez ^1,^ *, Benjamin de Haas ^2, 3, 4^, Chris A. Clark ^1^, Geraint Rees ^2, 3^, D. Samuel Schwarzkopf ^2, 3, 4^**

^1^ Institute of Child Health, University College London, London, UK
^2^ Institute of Cognitive Neuroscience, University College London, London, UK
^3^ Wellcome Trust Centre for Neuroimaging, University College London, London, UK

^4^ Experimental Psychology, University College London, London, UK

*** Correspondence:** Ivan Alvarez, Developmental Imaging and Biophysics Section, UCL Institute of Child Health, 30 Guilford Street, London, WC1N 1EH, UK.

Ivan.alvarez.11@ucl.ac.uk

1. **Video 1.**

Stimulus movie consisting of a dynamic, high-contrast pseudo-checkerboard carrier displayed through a size-invariant bar aperture traversing the visual field. The carrier varied in spatial frequency in time independently of the aperture position. Cardinal and oblique directions of transit were presented in separate acquisition runs, with both illustrated here. Aperture displacement is shown at approximately 10x speed of experimental presentation in the interest of brevity. A randomized attentional task at fixation is also not displayed.

1. **Video 2.**

Stimulus movie consisting of pseudo-checkerboard carrier displayed through an eccentricity-scaled bar aperture traversing the visual field. Bar width was scaled according to the inverse logarithm of the range of eccentricities displayed, in order to account for cortical magnification. Therefore, the central visual field was more finely sampled compared to the eccentric positions. Cardinal and oblique directions of transit were presented in separate acquisition runs, with both illustrated here. Aperture displacement is shown at approximately 10x speed of experimental presentation in the interest of brevity.

1. **Video 3.**

Stimulus movie consisting of pseudo-checkerboard carrier displayed through a simultaneous wedge and ring aperture, sampling polar angle and eccentricity, respectively. The ring component varied in eccentricity following a logarithmic function with 50% overlap between successive steps, while the wedge component was size-invariant. Each run contained a single direction of motion; with either a clockwise wedge and expanding ring, or a counter-clockwise wedge and contracting ring; both are illustrated here. Aperture displacement is shown at approximately 10x speed of experimental presentation in the interest of brevity.
